# A Cluster of *Peronospora parasitica 13-like* (*NBS-LRR*) Genes Is Associated with Powdery Mildew (*Erysiphe polygoni*) Resistance in Mungbean (*Vigna radiata*)

**DOI:** 10.3390/plants13091230

**Published:** 2024-04-29

**Authors:** Pitsanupong Waengwan, Kularb Laosatit, Yun Lin, Tarika Yimram, Xingxing Yuan, Xin Chen, Prakit Somta

**Affiliations:** 1 Department of Agronomy, Faculty of Agriculture at Kamphaeng Saen, Kasetsart University, Kamphaeng Saen 73140, Thailand; phitsanuphong.w@ku.th (P.W.); fagrkal@ku.ac.th (K.L.); tarikayimram@gmail.com (T.Y.); 2 Institute of Industrial Crops, Jiangsu Academy of Agricultural Sciences, Nanjing 210014, China; linyun881210@163.com (Y.L.); yxx@jaas.ac.cn (X.Y.); cx@jaas.ac.cn (X.C.)

**Keywords:** mungbean, *Vigna radiata*, powdery mildew, *Erysiphe polygoni*, *RPP13*

## Abstract

Powdery mildew (PM) caused by *Erysiphe polygoni* is an important foliar disease in mungbean (*Vigna radiata*). A previous study showed that QTL *qPMRUM5-2* is a major locus for PM resistance in mungbean accession RUM5 (highly resistant). Bioinformatics analysis revealed that flanking markers of the *qPMRUM5-2* covered a region of 1.93 Mb. In this study, we conducted fine mapping for the *qPMRUM5-2* using the F_2_ population of 1156 plants of the cross between Chai Nat 60 (CN60; highly susceptible) and RUM5. PM resistance evaluation was performed under field conditions using F_2:3_ lines grown in three different environments. QTL analyses consistently located the *qPMRUM5-2* to a 0.09 cm interval on linkage group 6 between InDel markers VrLG6-InDel05 and VrLG6-InDel10, which corresponded to a 135.0 kb region on chromosome 8 containing nine predicted genes of which five were NBS-LRR-type genes *Recognition of Peronospora parasitica 13-like protein* (*RPP13L*). Whole-genome re-sequencing of RUM5 and CN60 showed polymorphisms in four *RPP13L* genes predictively cause substantial amino acid changes, rendering them important candidate genes for PM resistance. The InDel markers VrLG6-InDel05 and VrLG6-InDel10 flanking to the *qPMRUM5-2* would be useful for marker-assisted breeding of PM resistance in the mungbean.

## 1. Introduction

Mungbean (*Vigna radiata* [L.] Wilczek) is an important legume crop grown in tropical and subtropical regions of Asia. It is widely grown in South and Southeast Asia, with a production area of more than 7.0 ha [1]. Mungbean seeds contain high proteins and carbohydrates of about 20–28% and 65–75%, respectively. The seeds are cooked and consumed or processed into several products, such as bean sprouts, starch, and protein isolates/concentrates. At present, mungbean has become a popular source of plant-based proteins. Major producers of mungbean include India, Myanmar, China, Indonesia, Pakistan, Bangladesh, Thailand, Cambodia, the Philippines, and Australia. The crop is now also popularly grown in several countries in Africa and America. Mungbean is fast growing, has an early maturity duration (60–75 days), and relative drought tolerance, and thus, it is generally grown as a sole crop following cereals such as rice, corn, and wheat or as a component in several cropping systems such as intercropping with sugar cane [1]. However, the average yield of mungbean is very low, being about 720 kg, although the potential yield of improved cultivars is about 2 tons/ha or higher.

Plant diseases are major factors causing low seed yield in the mungbean. Powdery mildew (PM), Cercospora leaf spot, and yellow mosaic virus are common foliar diseases of mungbean grown in Asia. PM disease caused by the fungus *Erysiphe polygoni* D.C. is an important foliar disease of mungbean [2]. It causes yield losses of up to 20–40% in susceptible cultivars [2]. This disease generally prevails in the cool–dry season of the tropical and subtropical region, which is a major season for the production of high-quality mungbean in some South and Southeast Asian countries [3]. Therefore, breeding of PM-resistant cultivars is one of the important goals in mungbean breeding programs. Mungbean germplasm identified for PM resistance and genetics of the resistance have been well documented. All the sources of PM resistance are from India. Among the resistant germplasm reported, the accession “RUM5” is the most interesting as it has been reported to be immune to PM disease in India [4,5], Taiwan [6], Thailand [7], and China (Xin Chen, personal observation).

A classical genetic study showed that the PM resistance in RUM5 is governed by two dominant loci: *Pm-1* and *Pm-2* [4]. Genotypes possessing both *Pm-1* and *Pm-2* are immune to the disease, while those possessing *Pm-1* are highly resistant, and those with *Pm-2* are moderately resistant [4]. Quantitative trait locus (QTL) analysis using F_2_ and backcross populations derived from crosses between RUM5 and PM-susceptible cultivar “Chai Nat 60” (CN60) revealed three QTLs, including *qPMRUM5-1*, *qPMRUM5-2*, and *qPMRUM5-3*, controlling the PM resistance in RUM5 [7]. *qPMRUM5-2* and *qPMRUM5-3* were major loci and were consistently identified in both F_2_ and backcross populations, while *qPMRUM5-1* was a minor locus and was only identified in one population [7]. Recently, Yundaeng et al. [3] showed that the gene *VrMLO12* (*LOC106773784*), a homolog to *AtMLO1* in *Arabidopsis thaliana*, on mungbean chromosome 9 encoding a Mildew Locus O protein is the candidate gene for the resistance at *qPMRUM5-3*. The MLO protein is well known for its involvement in powdery mildew disease in plants. The *qPMRUM5-2* was localized between simple sequence repeat (SSR) markers CEDG169 and CEDG121 on the mungbean linkage group (LG) 6 [7]. Comparative linkage analysis showed that *qPMRUM5-2* possibly be the same as the QTL *qPMR-1* conferring PM resistance in the mungbean breeding line “VC6468-11-1A” that was reported by Kasettranan et al. [7,8].

To date, molecular function(s) and mechanism(s) of *qPMRUM5-2* are not yet known. In this study, we report the molecular characterization of the *qPMRUM5-2* in RUM5, which will be useful for the genetic improvement in PM resistance in mungbean by molecular breeding. The objective of this study is to finely map the *qPMRUM5-2* and identify candidate genes for PM resistance.

## 2. Results

### 2.1. PM Disease Reaction in the F_2_ Population

**Figure 1:**
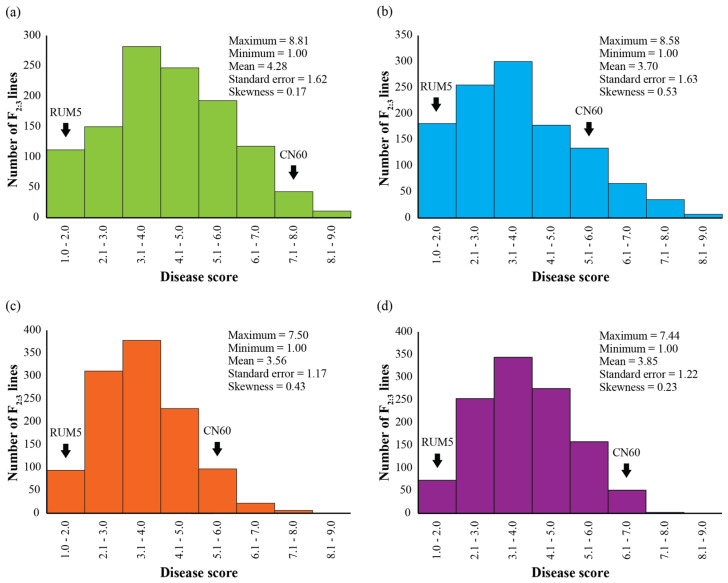
Frequency distribution of disease scores for resistance to powdery mildew disease caused by *E. polygoni* in 1156 F_2:3_ lines derived from the cross CN60 (susceptible) × RUM5 (resistant). The F_2:3_ lines and the parents were evaluated for resistance in three locations, viz. Nakhon Pathom (**a**), Chai Nat (**b**), and Petchabun (**c**). Average data of the disease scores from the three locations are shown in (**d**).

The reaction of PM resistance of the F_2_ population was evaluated using 1156 F_2:3_ lines grown in an augmented design in Chai Nat (CN), Nakhon Pathom (NP), and Petchabun (PB). The results of the PM resistance evaluation are summarized in Table 1. Disease scores of the F_2:3_ lines in these locations ranged between 1.00 and 8.81, 1.00 and 7.58, and 1.00 and 7.50 with means of 4.28, 3.70, and 3.56, respectively. Average disease scores of the F_2:3_ lines in the three locations (combined data) varied from 1.00 to 7.44 with a grand mean of 3.85. The parents, RUM5 and CN60, were shown to be highly different in PM resistance. The disease score in RUM5 was 1.00 at all three locations, while that in CN60 was 7.63, 5.77, and 5.72 in that order, with a mean across the locations of 6.37. The frequency distribution of disease scores of the F_2:3_ lines is shown in Figure 1.

### 2.2. Physical Location of the qPMRUM5-2 and Development of Novel DNA Markers for Fine Mapping

To determine the location of *qPMRUM5-2* on the reference genome of mungbean cultivar Sulv1, a BLASTN search was conducted to identify the location of the SSR markers CEDG169 and CEDG121 on LG6 that were previously identified as flanking markers to the *qPMRUM5-2*. The BLASTN search demonstrated that these SSR markers were physically on chromosome 8 at the positions 1,532,506 bp and 3,460,036 bp, respectively. Thus, the two markers covered a region of 1.928 Mb. In total, 191 new markers, including 161 SSR and 38 InDel markers, were developed from this 1.928 Mb region (Table S1). Marker screening in the RUM5 and CN60 showed that 98 markers revealed polymorphisms, while 14 SSR markers failed to amplify the DNA of these two mungbean accessions (Table S1). Nonetheless, 14 polymorphic markers (VrLG6-SSR02, VrLG6-SSR10, VrLG6-SSR15, VrLG6-SSR29, VrLG6-SSR40, VrLG6-SSR47, VrLG6-SSR54, VrLG6-SSR67, VrLG6-SSR78, VrLG6-SSR96, VrLG6-InDel04, VrLG6-InDel05, and VrLG6-InDel10) were selected and used to genotype the F_2_ population.

### 2.3. Fine Mapping of qPMRUM5-2

**Figure 2:**
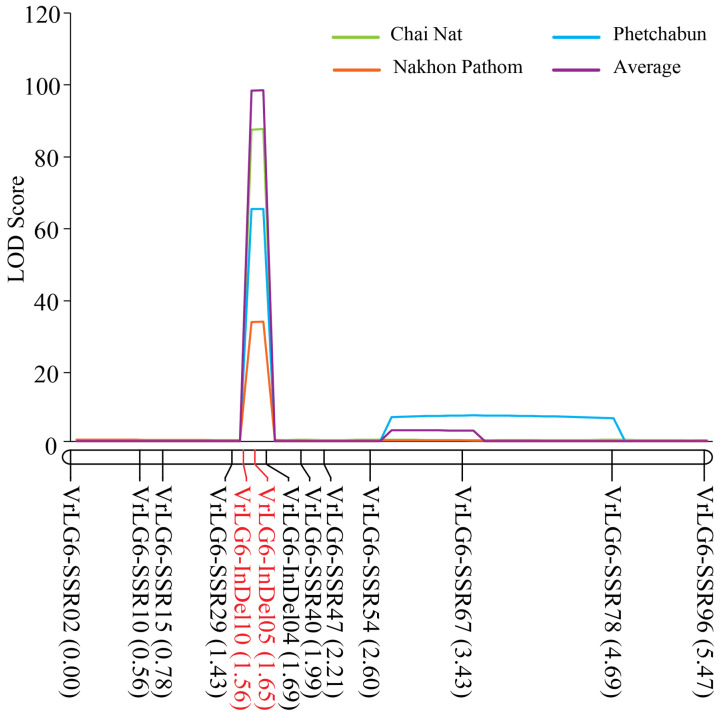
Logarithm of odds (LOD) graphs for the QTL *qPMRUM5-2* controlling powdery mildew disease resistance detected by inclusive composite interval mapping in the F_2:3_ population of the cross CN60 (susceptible) × RUM5 (resistant) grown in three locations. Markers flanking the *qPMRUM5-2* are highlighted in red.

A partial linkage map of LG6 for the F_2_ was constructed using 13 polymorphic markers developed in this study. The LG6 was 5.47 cM in length (Figure 2). QTL analysis for the PM resistance in different locations and combined data consistently located the *qPMRUM5-2* in the same location, which was 1.6 cM on the map between the InDel markers VrLG6-InDel10 and VrLG6-InDel05 (Table 1 and Figure 2). The *qPMRUM5-2* accounted for 29.61, 22.79, 13.22, and 32.40% of the disease score variation in CN, NP, PB, and the combined data, respectively. It expressed additive and dominant effects of 1.27 and 0.10 in CN, 1.11 and 0.00 in NP, 0.60 and 0.15 in PB, and 1.00 and 0.00 across the three locations, respectively.

### 2.4. Identification and Analysis of Candidate Genes for the qPMRUM5-2

**Figure 3:**
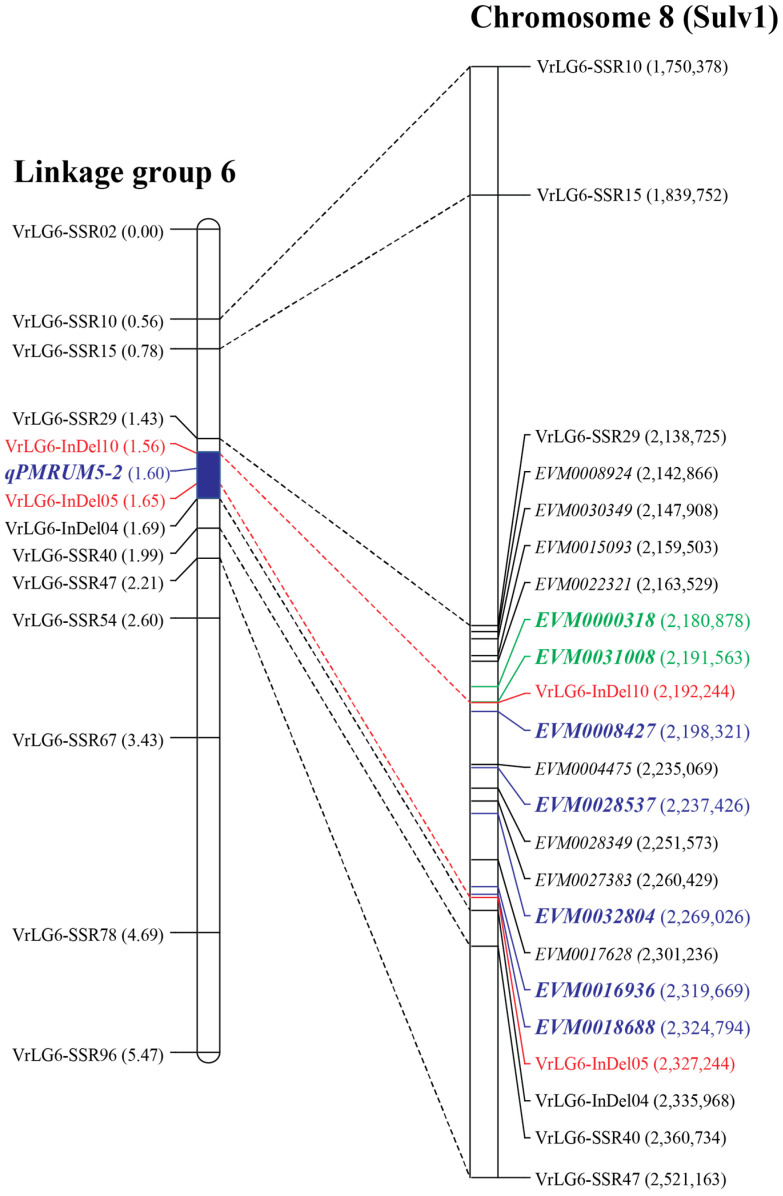
A comparative genome map illustrating the location of *qPMRUM5-2* detected by this study (**left**) and the candidate genes for the *qPMRUM5-2* on chromosome 8 of the mungbean reference genome (Sulv1) (**right**). Markers flanking the *qPMRUM5-2* are highlighted in red. The candidate genes are bolded and highlighted in blue or green.

We determined physical locations of the markers on the LG6 by BLASTN analysis. The results are illustrated in Figure 3. Orders of markers on LG6 perfectly agreed with their physical locations on the chromosome 8. Markers VrLG6-InDel10 and VrLG6-InDel05 flanking the *qPMRUM5-2* were only 135.0 Kb apart and were at the positions 2.192 Mb and 2.327 Mb on chromosome 8 in that order. There were nine predicted genes between the markers VrLG6-InDel10 and VrLG6-InDel05, in which five of them, including *EVM0008427*, *EVM0028537*, *EVM0032804*, *EVM0016936,* and *EVM0018688* encoded putative Recognition of *Peronospora parasitica* 13-like protein (RPP13L), a disease resistance protein. In addition, two other genes, *EVM0000318* and *EVM0031008*, encoding RPP13L protein, located very next to the 135.0 Kb QTL region. We considered these seven *RPP13L* genes as the candidate genes for the PM resistance at the *qPMRUM5-2*. They were designated *VrRPP13L3*, *VrRPP13L4*, *VrRPP13L5*, *VrRPP13L6*, *VrRPP13L7*, *VrRPP13L1,* and *VrRPP13L2*, respectively.

To investigate the sequence variations in the candidate genes between the resistant and susceptible parents, their genomes were re-sequenced. In total, 23.5 and 35.2 Gb raw re-sequencing data were obtained for CN60 and RUM5, respectively. The Q20 values of sequences of both CN60 and RUM5 reached >93%, while the Q30 values of sequences of both CN60 and RUM5 reached >83%. After aligning the sequences to the Sulv1 mungbean reference genome, 20.26 Gb and 29.04 Gb clean data of CN60 and RUM5, respectively, were used for further analysis. The data equaled genome coverage of ~38.7× and ~47.9×, respectively.

After filtration, a total of 3,876,952 high-quality SNPs and 375,548 InDels were identified between CN60 and RUM5. However, there existed 863 homozygous SNPs and 50 homozygous InDels in the 135.0 Kb QTL region (Tables S2 and S3). Of these SNPs, 428 (49.59%) were in the exons of six genes. Among these exonic SNPs, 471 (97.43%) presented in the five *VrRPP13L* genes: many nonsynonymous SNPs occurred in all the five *RPP13L1* genes, while two stop-gain SNPs existed in the *VrRPP13L4* gene, and three stop-gain SNPs presented in the *VrRPP13L5* and *VrRPP13L6* genes (Table 2, Table S2 and S3). In the case of the InDels, 11 (22.0%) of them were in the exons of four genes (Table 3). All except one InDel presented in three *RPP13L1* genes, including *VrRPP13L3*, *VrRPP13L5,* and *VrRPP13L6*. Five frameshift InDels and one stop-gain InDel occurred in the *VrRPP13L3* gene, while one each frameshift InDel presented in the genes *VrRPP13L5* and *VrRPP13L6* (Table 3). All the stop-gain SNPs and InDels occurred in RUM5.

In addition to the 135.0 Kb QTL region, the alignment also revealed 151 exonic SNPs and 4 exonic InDels in the *VrRPP13L2* gene (Table 2, Table 3, Table S2 and S3) located only 1.84 Kb away from the gene VrRPP13L3 (Figure 3). Of the 151 SNPs, 6 cause nonsynonymous SNPs, and 3 were splice-site SNPs. Among the four InDels, one was frameshift InDel, and one was stop-gain InDel.

## 3. Discussion

PM caused by *E. polygoni* in mungbean is among the plant biotic stresses that were subjected to gene mapping research at the beginning of the crop genomics era 30 years ago [9]. Nonetheless, due to the limited genomic tools and resources in this crop, little of the molecular basis of the PM resistance in mungbean is known at present. The QTLs *qPMRUM5-2* and *qPMRUM5-3* have been previously identified as major loci controlling the PM resistance in the mungbean accession RUM5 [7]. The molecular basis of the resistance conferred by the *qPMRUM5-3* was investigated recently in which *MLO12* was identified as the candidate gene for the resistance at this QTL [3].

Previously, the *qPMRUM5-2* was localized between the SSR markers CEDG169 and CEDG121 [7]. Based on the genome sequence of the mungbean cultivar Sulv1 [10], these two markers were nearly 2.0 Mb apart, and there were more than 150 genes in this region. In this study, by exploiting the Sulv1 genome sequence to develop new SSR and InDel markers and using a large F_2_ population for fine mapping, we successfully narrowed down the *qPMRUM5-2* to a genome region of 135.0 Kb on chromosome 8 of Sulv1 (Figure 3). Since QTL analysis using data of PM resistance obtained from three different environments consistently identified the *qPMRUM5-2* at the same position (Table 1 and Figure 2), the position of the *qPMRUM5-2* detected in this study is highly accurate. Based on the reference genome sequence of Sulv1, the *qPMRUM5-2* was located in a genomic region containing a cluster of seven *VrRPP13L* genes (*VrRPP13L1—VrRPP13L7*). These genes spanned a region of 146.4 Kb (Figure 3), and they were considered the candidate genes for the powdery mildew resistance in RUM5.

*VrRPP13L* is a homolog to the *RPP13* gene, which is a plant disease resistance gene (*R* gene) that encodes proteins with nucleotide-binding site (NBS) and leucine-rich repeat (LRR) domains (NBS–LRR proteins). In *Arabidopsis thaliana* L., *RPP13* is a simple locus conferring a broad resistance to downy mildew disease caused by the fungus *Hyaloperonospora parasitica* (Gaum.) (synonym *Peronospora parasitica* (Pers. ex. Fr.)) by providing hypersensitive response [11]. *RPP13L* genes have been shown to be associated with resistance to several diseases in various crops. For example, resistance to powdery mildew caused by *Bgt* in wheat (*Aegilops biuncialis* Vis.) cultivar [12] and by *Blumeria graminis* f.sp. *tritici* (Bgt) in barley (*Hordeum vulgare* L.) [13,14], root-knot disease caused by *Meloidogyne incognita* in common bean (*Phaseolus vulgaris* L.), and clover rot disease caused by *Sclerotinia trifoliorum* Bain & Essary in red clover (*Trifolium pratense* L.) [15]. Interestingly, a *RPP13L* gene, *Phavu_004G036200g*, has been identified as a candidate gene for a locus conferring resistance to powdery mildew caused by *E. polygoni* in common bean (*Phaseolus vulgaris* L.), a legume species genetically close to mungbean [16]. We conducted BLASTP analysis and found that *Phavu_004G036200g* is an ortholog to the *VrRPP13L* genes identified in our study.

Since the seven *VrRPP13L* genes are clustered in a small region of only 146.4 kb, and six of the genes (*VrRPP13L2*—*VrRPP13L7*) showed exonic sequence polymorphisms that may alter the function of RPP13L proteins between the mapping parents (Supplementary Table S2), it will be difficult to determine which one of them, all of them, or any combinations of them provide powdery mildew resistance in RUM5. Additional study using a large population size to further identify the causal gene(s) for the resistance at this gene cluster is necessary. Nonetheless, the diversity and evolution of the *R* genes and their loci (*R* genes cluster) in plants can stem from recombination, duplications/deletions, and positive selection [17,18]. Due to the fact that the sequences of individual *VrRPP13L* genes in the *R*-genes cluster identified in this study are highly homologous, those *VrRPP13L* may have evolved via duplications. In addition, clustering of those *VrRPP13L* genes can lead to the evolution of novel resistance specificities to *E. polygoni* by unequal crossing over and/or gene conversion. It is noteworthy that as compared to the Sulv1 reference genome, 88.4% of the sequence polymorphisms found in the seven *VrRPP13L* genes between RUM5 and CN60 were contributed by RUM5 (Supplementary Table S2). The high diversity of *VrRPP13L* genes in RUM5 may provide the high and broad resistance of this mungbean germplasm against the fungus *E. polygoni.*

RUM5 is a valuable genetic resource for breeding of PM resistance in mungbean because it showed high and stable resistance in India, Thailand, Taiwan, and China. *qPMRUM5-2* and *qPMRUM5-3* are the QTLs for the PM resistance in RUM5 [7]. In this study, we demonstrated that a cluster of *VrRPP13L* genes, *R*-gene loci, is associated with the resistance at the *qPMRUM5-2*. Yundaeng et al. (2020) [3] showed that *VrMLO12* encoding a protein belonging to MLO clade II is responsible for the resistance at the *qPMRUM5-3*. *MLO* is known as a susceptibility gene (*S*-gene), whose loss of function leads to recessively inherited resistance [19]. Hence, it appears that the high and broad resistance in RUM5 is a combined effect of polymorphisms in both *S*-gene and *R*-gene. The *VrMLO12* and the *VrRPP13L* genes cluster in RUM5 would be useful for genetic improvement of PM resistance in mungbean. An InDel marker, VrMLO12-Indel3, specific to the *VrMLO12* gene from RUM5, was developed [3]. The SSR marker VrLG6-SSR29, together with InDel markers VrLG6-InDel-05 and VrLG6-InDel-10, flanked the *VrRPP13L* gene cluster (Figure 3). These four markers can be utilized for breeding PM resistance in mungbean by pyramiding the *VrRPP13L* genes cluster and *VrMLO12* gene from RUM5 into susceptible mungbean cultivar(s) via marker-assisted selection.

## 4. Materials and Methods

### 4.1. Plant Materials and DNA Extraction

An F_2_ population comprising 1156 plants was developed from a cross between RUM5 (male parent) and CN60 (female parent). CN60 is a cultivar from Thailand and is highly susceptible to PM, whereas RUM5 is immune or highly resistant to PM [3,7]. In total, 1156 F_2_ plants and their parents were grown under a field condition and self-pollinated to produce F_2:3_ seeds. The total genomic DNA of the F_2_ plants and the parental plants was extracted from young leaves following a CTAB method described by Lodhi et al. [20]. The DNA was assessed for quality and quantity using a NanoDrop™ 2000 spectrophotometer (Thermo Fisher Scientific, Waltham, MA, USA).

### 4.2. Evaluation for Powdery Mildew Disease

The F_2:3_ lines and the parents were evaluated for PM resistance under field conditions in three locations of Thailand, viz. (1) Chai Nat Field Crops Research Center, Sappaya, Chai Nat province, (2) Kasetsart University, Kamphaeng Saen Campus, Kamphaeng Saen, Nakhon Pathom province, and (3) Ta Daeng, Nong Phai, Petchabun province. The evaluation at Chai Nat was conducted from late December 2020 to early March 2021, while the evaluation at Nakhon Pathom and Petchabun was carried out from late December 2021 to early March 2022. At all locations, the evaluation was performed using augmented design [21]. RUM5 and CN60 were used as “check” cultivars. CN60 was planted around the field as a natural spreader of the PM disease. The F_2:3_ lines and the parental lines were grown in a single row of 1.5 m with two plants per hill (24 plants/row). The intra-row and inter-row spacings were 12.5 cm and 50 cm, respectively.

At 20, 25, and 30 days after planting (DAP), plants were inoculated with spore suspensions prepared from PM-infected leaves. At 60 DAP, 10 plants of each entry were randomly selected and visually scored for PM disease reaction using a scale of 1 to 9, where 1 had no disease infection; 3 had 1–25% leaf area infected; 5 had 26–50% leaf area infected, 7 had 51–75% leaf area infected, and 9 had 76–100% leaf area infected [7,9]. The disease scoring was carried out by three members of staff.

### 4.3. Determination of the Physical Position of qPMRUM5-2 and Development of Novel DNA Markers for Fine Mapping

Since the *qPMRUM5-2* was previously mapped to a region between SSR markers CEDG169 and CEDG121 [7], we identified chromosomal locations of the two markers by conducting a BLASTN search of their primer sequences against the genome sequence of mungbean cultivar Sulv1 [10] in order to determine the location of *qPMRUM5-2* on the reference genome. The genome sequence between markers CEDG169 and CEDG121 was detected for SSRs using SSRIT [22], and then, primers were designed for the SSRs utilizing Primer3 [23]. The primers were screened for polymorphism between RUM5 and CN60. Polymerase chain reaction (PCR) was conducted in a total volume of 10 μL containing 5 ng of genomic DNA, 0.2 mM dNTPs, 1 U *Taq* DNA polymerase 2 mM MgCl2, and 1 × *Taq* buffer (Thermo Fisher Scientific Inc., Waltham, MA, USA), and 2.5 μM each of forward and reverse primers. Amplification was performed using SimpliAmp™ Thermal Cycler (Thermo Fisher Scientific, Waltham, MA, USA) programmed as follows: 94 °C for 2 min followed by 35 cycles of 94 °C for 30 s, 55 °C for 30 s, 72 °C for 30 s, and 72 °C for 10 min. PCR products were run on 5% polyacrylamide gel electrophoresis. DNA bands were visualized by silver staining. In total, 24 markers showing clear polymorphism were used to genotype all the F_2_ plants.

In addition, InDel markers were developed based for the InDel polymorphisms detected between CN60 and RUM by whole-genome resequencing and alignment (see Section 4.5). Primers design and polymorphisms screening for the InDel markers were performed using the same procedures described above for the SSR markers.

### 4.4. Linkage and QTL Analyses

The linkage map for the F_2_ population was constructed using the software QTL IciMapping 4.2 [24]. Markers were grouped with logarithm of odds (LOD) values of 5.0 and then ordered using REcombination Counting and ORDering (RECORD) method [25]. Distances between markers were calculated using Kosambi’s mapping function [26].

QTL for PM resistance was localized on the linkage map using the inclusive composite interval mapping method (ICIM) [27] by QTL IciMapping 4.2. A significant LOD threshold for declaring QTL was calculated by a 1000 permutation test at the probability of 0.001.

### 4.5. Candidate Genes Analysis

Markers flanking the *qPMRUM5-2* were used to BLAST against the reference genome of mungbean cultivar Sulv1 [10] to determine of the QTL region. Genes located within and near the QTL region were considered candidate genes for the PM resistance in RUM5. In order to discover the DNA polymorphism(s) in the candidate genes, genomes of RUM5 and CN60 were re-sequenced as described by Arai-Kichise et al. [28]. In brief, Illumina pair-end sequencing (PE150) was performed. At least 1μg DNA of RUM5 and CN60 was used for the construction of paired-end sequencing libraries with insert sizes of ~450 bp. Purified DNA is sheared into smaller fragments with a desired size using Adaptive Focused Acoustics^®^ technology (Covaris, Woburn, MA, USA) and T4 DNA polymerase was applied to generate blunt ends. After adding an ‘A’ base to the 3′ end of the blunt phosphorylated DNA fragments, adapters are ligated to the ends. The desired fragments were purified via gel electrophoresis and then selectively enriched and amplified by PCR. Index tags were introduced into the adapter at the PCR stage, followed by a library quality test. The amplified products were quantified by TBS-380 Fluorometer (Promega, Madison, WI, USA). Subsequently, the paired-end libraries were sequenced using Illumina NovaSeq PE150 (Illumina, San Diego, CA, USA) by Shanghai Biozeron Biotechnology Co., Ltd. (Shanghai, China). After sequencing, the raw paired-end reads were trimmed and quality controlled by Trimmomatic (http://www.usadellab.org/cms/?page=trimmomatic, accessed on 28 February 2023) with default parameters.

High-quality sequencing reads were aligned against the reference genome of Sulv1 [10] using BWA (http://bio-bwa.sourceforge.net, accessed on 20 February 2023) software. Sequence Alignment/Map (SAM) format files [29] were imported into SAMtools [29] (http://samtools.sourceforge.net, accessed on 28 February 2023) for sorting and merging and into Picard (http://broadinstitute.github.io/picard/, version 1.92, accessed on 28 February 2023) to remove duplicated reads. BAM file was used to identify SNPs and short InDel (1–10 bp) using Genome Analysis Toolkit (GATK) version 4.1.2.0 (McKenna et al., 2010) [30] with “UnifiedGenotyper” function (https://gatk.broadinstitute.org, accessed on 28 February 2023).

## Figures and Tables

**Table 1:** Details of the *qPMRUM5-2* on linkage group 6 conferring to powdery mildew disease caused by *Erysiphe polygoni*. The *qPMRUM5-2* was detected using an F_2_ population of 1152 individuals derived from the cross-Chai Nat 60 × RUM5.

Location	Year	Position (cM)	Flanking Marker	LOD Score	PVE ^a^	Add ^b^	Dom ^c^
Chai Nat	2019	1.6	VrLG6-InDel10—VrLG6-InDel05	87.71	29.61	1.27	0.10
Phetchabun	2020	1.6	VrLG6-InDel10—VrLG6-InDel05	65.52	22.79	1.11	0.00
Nakhon Pathom	2020	1.6	VrLG6-InDel10—VrLG6-InDel05	34.20	13.22	0.60	0.15
Combined		1.6	VrLG6-InDel10—VrLG6-InDel05	98.53	32.40	1.00	0.00

^a^ PVE = percentage of variance explained by the QTL; ^b^ Add = additive gene effect; ^c^ Dom = dominant gene effect.

**Table 2:** Summary of single-nucleotide polymorphisms identified between Chai Nat 60 (CN60) and RUM5 in *RPP13L* genes located in and near the *qPMRUM5-2* region.

Gene	Region	Number of SNPs	Types of Mutation
*EVM0008427*	upstream	5	-
(*VrRPP13L3*)	exonic	229	nonsynonymous SNP (158) and synonymous SNP (71)
	3′UTR	9	-
	intergenic	71	-
*EVM0028537*	upstream	4	-
(*VrRPP13L4*)	exonic	51	nonsynonymous SNP (31), synonymous SNP (18) and stop-gain SNP (2)
	intronic	39	
*EVM0032804*	upstream	1	-
(*VrRPP13L5*)	5′UTR	3	-
	exonic	77	nonsynonymous SNP (52), synonymous SNP (22), and stop-gain SNP (3)
	intergenic	53	-
*EVM0016936*	upstream	20	-
(*VrRPP13L6*)	exonic	35	nonsynonymous SNP (21), synonymous SNP (11), and stop-gain SNP (3)
	downstream	21	-
	intergenic	22	-
*EVM0018688*	upstream	49	-
(*VrRPP13L7*)	5′UTR	1	-
	exonic	25	nonsynonymous SNP (16) and synonymous SNP (9)
	3′UTR	3	-
	downstream	21	-
	intergenic	75	-
*EVM0000318* (*VrRPP13L1*)	intergenic	9	-
*EVM0031008* (*VrRPP13L2*)	exonic	151	nonsynonymous SNP (102), synonymous SNP (46), and splicing-relevant SNP (3)
	intronic	75	-
	downstream	23	-

**Table 3:** Summary of insertions/deletions (InDels) identified between Chai Nat 60 (CN60) and RUM5 in *RPP13L* genes located in and near the *qPMRUM5-2* region.

Gene	Region	Number of InDels	Types of Mutation
*EVM0008427*	exonic	8	nonframeshift InDel (2), frameshift InDel (5) and stop-gain InDel (1)
(*VrRPP13L3*)	intronic	1	-
	3′UTR	1	-
*EVM0032804*	exonic	1	frameshift InDel
(*VrRPP13L5*)	intergenic	5	-
*EVM0016936*	upstream	2	-
(*VrRPP13L6*)	exonic	1	frameshift InDel
	downstream	2	-
	intergenic	4	-
*EVM0018688*	upstream	10	-
(*VrRPP13L7*)	downstream	5	-
	intergenic	8	-
*EVM0031008*	exonic	4	nonframeshift InDel (2), frameshift InDel (1) and stop-gain InDel (1)
(*VrRPP13L2*)	intronic	7	-
	downstream	2	-

## Data Availability

The whole-genome resequencing data generated in this study were deposited to the National Center for Biotechnology Information (https://www.ncbi.nlm.nih.gov) (Project accession number “PRJNA977077”). Other datasets generated during and/or analyzed during the current study are available from the corresponding author upon reasonable request.

## Supplementary Materials

The following supporting information can be downloaded at https://www.mdpi.com/article/10.3390/plants13091230/s1. Table S1: Details of SSR and Indel markers used in this study; Table S2: Single-nucleotide polymorphisms between Chai Nat 60 (CN60) and RUM5 in *RPP13L1* genes located in and near the *qPMRUM5-2* region; Table S3: InDel polymorphisms between Chai Nat 60 (CN60) and RUM5 in *RPP13L1* genes located in and near the *qPMRUM5-2* region.
